# Chromosome Y pericentric heterochromatin is a primary target of HSF1 in male cells

**DOI:** 10.1007/s00412-021-00751-2

**Published:** 2021-02-06

**Authors:** Jessica Penin, Solenne Dufour, Virginie Faure, Sabrina Fritah, Daphné Seigneurin-Berny, Edwige Col, André Verdel, Claire Vourc’h

**Affiliations:** 1 RNA and Epigenetics Team, Institute for Advanced Biosciences (IAB), Centre de Recherche UGA / Inserm U 1209 / CNRS UMR 5309, La Tronche BP170, 39042 Grenoble Cedex, France; 2 Department of Oncology, Luxembourg Institute of Health, NORLUX Neuro-Oncology Laboratory, L1526 Luxembourg, Luxembourg

**Keywords:** HSF1, Heterochromatin, ncRNA, nSB, Human

## Abstract

The heat shock factor 1 (HSF1)-dependent transcriptional activation of human pericentric heterochromatin in heat-shocked cells is the most striking example of transcriptional activation of heterochromatin. Until now, pericentric heterochromatin of chromosome 9 has been identified as the primary target of HSF1, in both normal and tumor heat-shocked cells. Transcriptional awakening of this large genomic region results in the nuclear accumulation of satellite III (*SATIII*) noncoding RNAs (ncRNAs) and the formation in *cis* of specific structures known as nuclear stress bodies (nSBs). Here, we show that, in four different male cell lines, including primary human fibroblasts and amniocytes, pericentric heterochromatin of chromosome Y can also serve as a unique primary site of HSF1-dependent heterochromatin transcriptional activation, production of *SATIII* ncRNA, and nucleation of nuclear stress bodies (nSBs) upon heat shock. Our observation suggests that the chromosomal origin of *SATIII* transcripts in cells submitted to heat shock is not a determinant factor as such, but that transcription of *SATIII* repetitive units or the *SATIII* ncRNA molecules is the critical element of HSF1-dependent transcription activation of constitutive heterochromatin.

**Supplementary Information:**

The online version contains supplementary material available at 10.1007/s00412-021-00751-2.

## Introduction

Cell exposure to proteotoxic stress, such as heat shock, induces major and transient changes in gene expression. This response is under the control of a key transcription factor, named heat shock factor 1 (HSF1) (Akerfelt et al. 2010a; Li et al. 2017; Barna et al. 2018). Upon heat shock, HSF1 becomes hyperphosphorylated, trimerizes, and transactivates stress-induced genes, including heat shock protein (*HSP*) coding genes (Akerfelt et al. 2010a). In addition, a transcriptional activation of pericentric heterochromatin enriched in satellite III repeated DNA sequences has also been described (Jolly et al. 2004; Rizzi et al. 2004; Eymery et al. 2009). *SATIII* sequences are characterized by repetitive GGAAT motifs organized in tandem and head to tail orientation. Pericentric heterochromatin contains a large number of transcription units covering several megabases. This unique configuration allows an in situ characterization of the different actors involved in the transcriptional activation of these sequences. Likewise, upon heat shock, the recruitment of HSF1, histone acetyltransferases (Goenka et al. 2016; Col et al. 2017), and RNA polymerase II (RNAPII) to pericentric heterochromatin lead to the formation of stress-specific structures known as nuclear stress bodies (nSBs) (Biamonti and Vourc’h 2010). nSBs can be easily detected by immunofluorescence using anti-HSF1 or anti-acetylated histone antibodies (Rizzi et al. 2004; Jolly et al. 2004). *SATIII* transcripts are ncRNAs, heterogeneous in size with a large fraction in the 2 to 5 kb range (Jolly et al. 2004; Rizzi et al. 2004). Once transcribed, they accumulate at transcription sites, suggesting a role for these transcripts in *cis,* possibly linked to the maintenance and/or reformation of heterochromatin in stressed cells. Additionally, recruitment of various splicing factors to nSBs and their interaction with *SATIII* transcripts suggest that *SATIII* transcripts may also undergo post-transcriptional maturation or that this recruitment may contribute to a reprogramming of nuclear functions through their transient sequestration at nSBs (Biamonti and Vourc’h 2010; Metz et al. 2004; Ninomiya et al. 2020).

In normal heat-shocked female human cells, nSBs are detected at heterochromatic regions of chromosome 9 (9q12 locus) (Jolly et al. 2002). In contrast, in heat-shocked female and male cancer cells, several secondary nSBs are also detected. Furthermore, the number and intensity of these secondary nSBs increases with the level of HSF1 expression within the cells (Eymery et al. 2010). Based on in vitro reconstitution of nSBs, obtained by incubating metaphase chromosome spreads with purified active HSF1, a number of chromosomes, all containing *SATII* and *SATIII* sequences, have been identified as HSF1 targets, including chromosome Y (Eymery et al. 2010). Likewise, in stressed rodent/human hybrid cell lines containing a unique human chromosome, nSBs are observed when *SATII*/*SATIII*-containing chromosomes are present, confirming their potential to form nSBs in an independent and autonomous fashion (Eymery et al. 2010). Yet, whether they may exert functions similar to the primary nSBs linked to the 9q12 locus remains unknown.

Here, we identify pericentric heterochromatin of chromosome Y as a primary target of HSF1, and not only as a HSF1 secondary target, in both cancer and primary cell lines. Moreover, our observation that nSBs form on chromosome Y and not on chromosome 9 in stressed HT1080 cells suggests that the protective role of nSBs goes through the act of transcription of *SATIII* DNA repeats and/or the selective recruitment of transcription and RNA processing factors, regardless of the very nature of the chromosome on which they form.

## Results

### nSBs formation on chromosome Y in heat-shocked cells

**Fig. 1:**
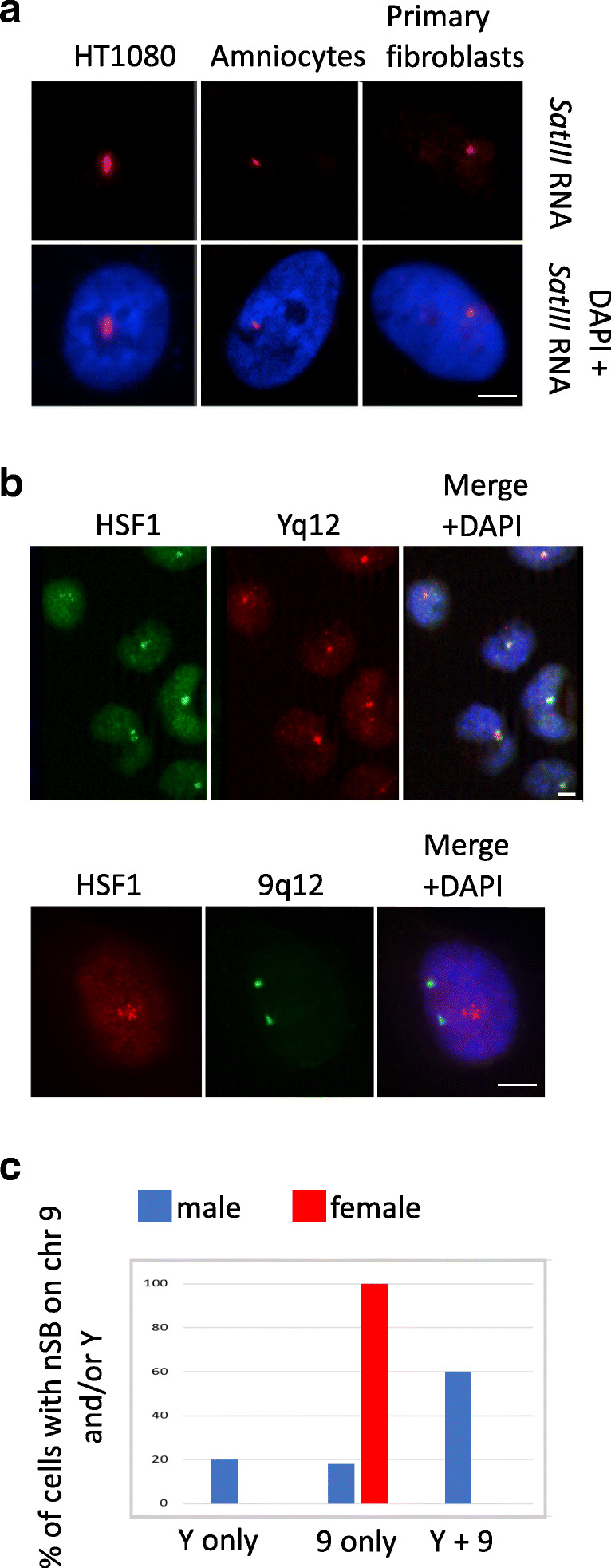
Heat-shocked HT1080 cells display a unique HSF1 foci on chromosome Y. **a** Unique *SATIII* RNA foci are observed in the nucleus of cell lines of male origin (bar = 5 μm). *SATIII* RNA (red signals) are detected by RNA FISH with a fluorescent oligonucleotide recognizing (GGAAU)n motifs, on male cells of different origin (HT1080 cells, amniocytes and primary fibroblasts). **b** HSF1 detected by immuno-fluorescence in HT1080 cells colocalizes with the Yq12 locus and not with the 9q12 locus detected by DNA FISH. Upper image Yq12 locus (red signals), HSF1 (green signal) (bar = 5 μm). Lower image 9q12 locus (green signal), HSF1 (red signal) (bar = 5 μm). **c** Graph giving the percentage of nSBs present on chromosome 9, chromosome Y, or both, in normal fibroblasts. HSF1 foci were codetected by immunofluorescence and FISH in human primary stressed male and female fibroblasts (total of 200 nuclei analyzed on three independent analyses)

Until now, the q12 locus of chromosome 9 has always been reported as the main site for nSBs formation in stressed cells, i.e., the locus where nucleation of HSF1 foci is initiated overtime, suggesting that the accumulation of HSF1 at the 9q12 locus could have a role specifically related to the chromosome 9 itself. However, we uncovered that male cell lines, in particular fibrosarcoma HT1080 cells, often display a single *SATIII* RNA nuclear foci, which is not consistent with nSBs formation at 9q12 since these diploid cells possess two 9q12 loci (Fig. 1a). This prompted us to further characterize the chromosomal origin of these unique foci. Combining immunofluorescence to detect HSF1 and fluorescence in situ hybridization (FISH) to detect chromosome Y-specific *SATIII* sequences, we found that, in HT1080 male stressed cells, HSF1 forms primary foci on chromosome Y and not on chromosome 9 (Fig. 1b). Interestingly though, HSF1 still has the capacity to bind to the 9q12 loci in in vitro HSF1-binding assays performed with purified HSF1 on mitotic chromosomes from HT1080 cells (Fig. S1). Similar to HT1080 cells, primary HSF1 foci forming on chromosome Y (and not on chromosome 9) were also observed in the H460 cell line of lung origin (Fig. S2). In contrast to HT1080 and H460 cells, HSF1 foci were found to match either with chromosome 9, chromosome Y, or both chromosomes in normal male fibroblasts. Indeed, in these cells, the same percentage of cells displaying a primary nSB on chromosome 9 (19%) or chromosome Y (21%) was observed, with a large percentage of cells displaying signals on both chromosomes 9 and Y (60%), suggesting that, in normal male fibroblasts, both chromosomes 9 and Y are primary targets for HSF1 (Fig. 1c).

### Formation of nSBs at 9q12 locus in HT1080 cells depends on the cellular level of HSF1

**Fig. 2:**
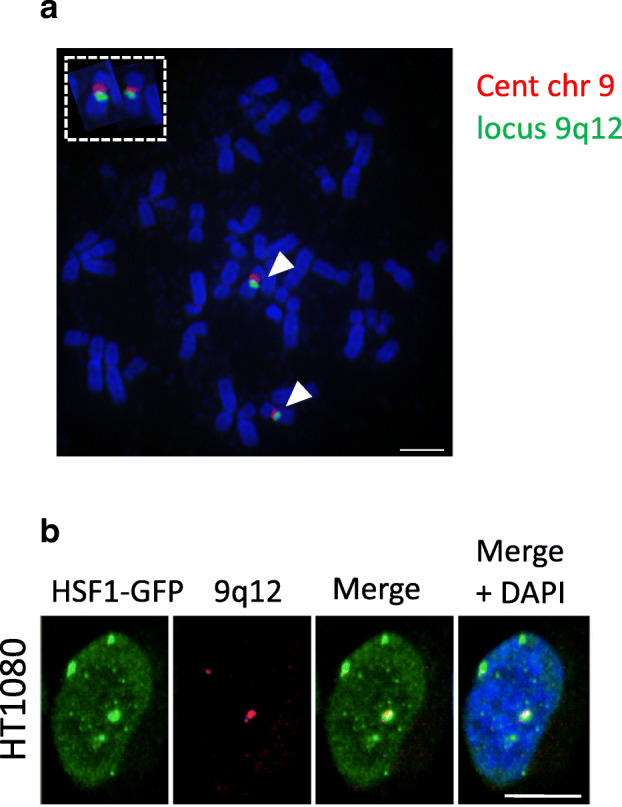
HSF1 overexpression leads to its binding to pericentric regions of chromosome 9 in HT1080 cells. **a** HT1080 cells do not lack chromosome-9-specific *SATIII* genomic sequences. Simultaneous detection of *SATIII* sequences from chromosome 9 pericentric regions detected with the pHuR98 probe (red signal) and of alphoid sequences from chromosome 9 centromeric regions with the pMR9A (green signal) on mitotic spreads from HT1080 cells. The two chromosomes 9 are also enlarged (bar = 5 μm). **b** HT1080 cells transiently transfected with a HSF1-GFP expression vector display HSF1 foci (green signals) at the 9q12 loci detected by FISH (red signal) (bar = 10 μm)

We further investigated why, in contrast to normal male fibroblast cells, pericentric heterochromatin of chromosome Y is primarily targeted by HSF1 in HT1080 cells. Sequencing of a HSF1 cDNA sequence prepared from HT1080 cells did not show any specific feature in the coding sequence that could have explained HSF1 preferential targeting on chromosome Y in these cells (data not shown). We also confirmed that HT1080 cells do contain chromosome 9-specific *SATIII* sequences by conducting DNA FISH experiment with a probe specific for chromosome 9 *SATIII* sequences on metaphase spreads (Fig. 2a). Noticeably, we found that the formation of nSBs on chromosome 9 occurs in HT1080 male cells when these cells are transiently transfected with a plasmid expressing the HSF1-GFP fusion protein (Fig. 2b). This later result indicates that, in HT1080 cells, an increase in expression of HSF1 is sufficient to restore the formation of nSBs at chromosome 9. Since the level of endogenous HSF1 in HT1080 cells is similar in HeLa cells (Fig. S3), and since HSF1 foci at chromosome 9 are restored in HT1080 cells when overexpressing HSF1, the lack of HSF1 binding on chromosome 9 likely reflects the existence of a preferential binding of HSF1 on chromosome Y, at least in these cells.

### Yq12 *SATIII* DNA repeats are required for formation of nSBs on chromosome Y

**Fig. 3:**
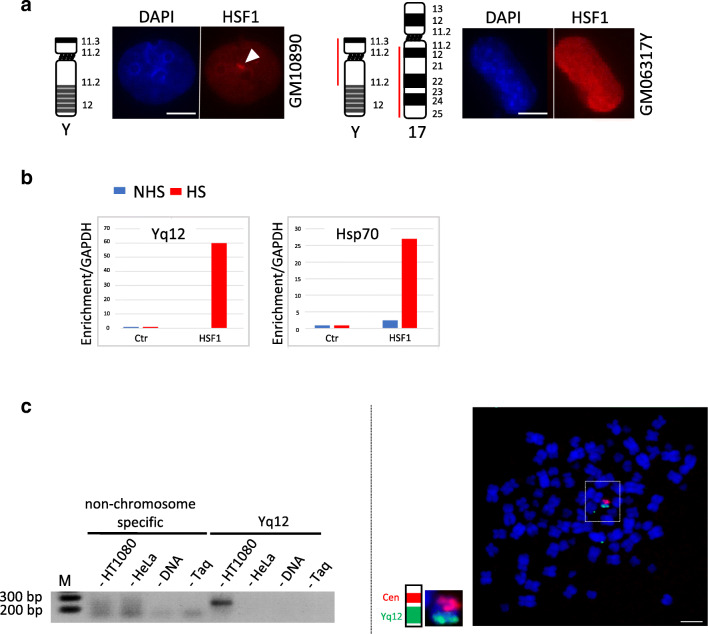
HSF1 binds chromosome Y *SATIII* sequences upon heat shock. **a** HSF1 detection by immunofluorescence on somatic hamster/human hybrid cell lines containing either chromosome Y (GM10890 cells) or only the Yp->Yq11::17q12->17qter (GM06317Y cells) region of chromosome Y. HSF1 foci were only detected in hybrid cell lines containing the Yq12 locus (bar = 10 μm). **b** ChIP experiments with anti-HSF1 antibody performed on unstressed (NHS) and heat shock (HS) HT1080 cells (anti-IgG antibody were used as a control, Ctr). PCR amplifications were performed with primers specific to either chromosome-Y-specific *SATIII* sequences or to the *HSP*70 gene. **c** The enrichment of genomic *SATIII* sequences of chromosome Y origin in anti-HSF1 ChIP experiments was assessed. First (left image), a PCR product, here analyzed by electrophoresis, was only obtained with genomic DNA from stressed HT1080 cell line of male origin and not with genomic DNA from stressed HeLa cells of female origin. Controls are shown in which the amplification reactions were performed with no DNA or no DNA polymerase (Taq). A PCR product is only obtained with genomic DNA of male origin. Second (right image), the PCR product obtained from HT1080 cells was labeled and used for a DNA FISH experiment on metaphase spreads from HT1080 cells (green signal). A centromeric probe was also used to identify chromosome Y (red signal) (bar = 5 μm). The PCR product hybridizes to chromosome Y when used as a probe in a DNA FISH experiment

Binding of HSF1 on chromosome Y has been reported at multicopy genes in mouse (Akerfelt et al. 2010). We therefore tested whether the formation of nSBs on chromosome Y depends on the presence of *SATIII* sequences, and not just on the presence of other chromosome Y repetitive elements (Akerfelt et al. 2010). This was investigated in two ways. We first tested the presence of nSBs in two different human/hamster somatic hybrid cell lines, either containing the entire chromosome Y (GM10890 cells) as the only human material or containing a truncated chromosome Y depleted of its Yq12 band and thus depleted of its *SATIII* sequences (GM06317Y cells) (Fig. 3a). Since HSF1 does not form foci in hamster cell lines, human/hamster hybrid cells represent powerful tools to test the importance of human genomic sequences in recruiting HSF1 (Eymery et al. 2010). Importantly, in contrast to GM10890 cells, in which HSF1 forms foci upon heat shock, no HSF1 foci were detected in stressed GM06317Y cells, suggesting that SatIII DNA repeats present on chromosome Y are absolutely required for the recruitment of HSF1 and the formation of nSBs (Fig. 3a). We next directly searched for the presence of heat shock element (HSEs) (Kroeger and Morimoto 1994; Trinklein et al. 2004) within chromosome Y-specific genomic *SATIII* sequence. In agreement with a direct binding of HSF1 to the *SATIII* region of chromosome Y, four perfect HSEs were detected on a 3564-bp-long genomic *SATIII* sequence localizing within the Yq12 locus (GenBank: X06228.1, Nakahori et al. 1986). This prompted us to examine whether a region of chromosome Y containing a putative HSF1 binding site is indeed targeted by HSF1 upon heat shock, by conducting chromatin immuno precipitation (ChIP) experiments with an anti HSF1 antibody (Fig. 3b). Chromosome Y-specific *SATIII* primers were designed, delineating a 154-bp region positioned 241 bp upstream of a putative heat shock element (Fig. S4a and b). Activation of HSF1 in heat-shocked HT1080 cells was monitored by western blot using an anti-HSF1 antibody (Fig. S4c**)** while binding of HSF1 to the promoter of the HSP70 gene was used as a positive control of ChIP experiment (Fig. 3b, right). As expected, we only found enrichment of Y-chromosome *SATIII* sequences in ChIP experiments conducted on male genomic DNA (HT1080 cells) but not from HeLa cells, used as a negative control (Fig. 3c), left. To check for the specificity of the PCR product obtained on HT1080 cells, this PCR product was further used as a probe in a FISH experiment on male mitotic chromosome spreads from HT1080 cells. Importantly, a unique hybridization signal was observed on chromosome Y (Fig. 3c, right). Thus, collectively, these results strongly suggest that upon heat shock, HSF1 binds HSE elements interspersed within the *SATIII* sequences of chromosome Y to form nSBs in HT1080 cells.

### Splicing factor SRSF1 is recruited to active *SATIII* transcription sites in HT1080-stressed cells

**Fig. 4:**
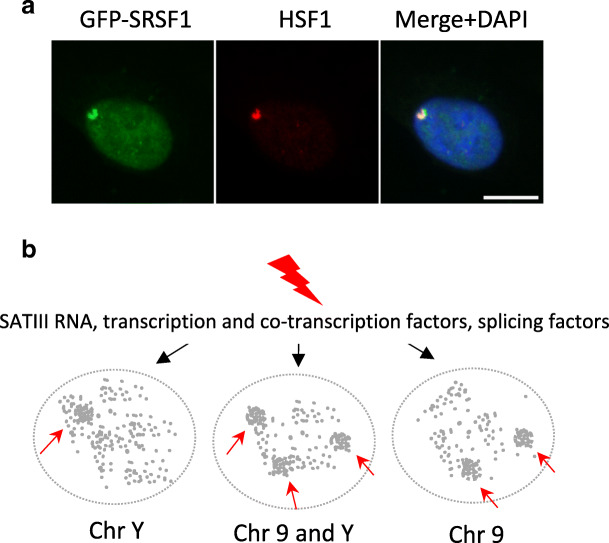
SRSF1 is recruited to chromosome-Y-specific active *SATIII* transcription sites in human cells upon heat shock. **a** SRSF1-GFP splicing factors form unique foci (green signal) colocalizing with HSF1 (red signal) detected by immunofluorescence in stressed HT1080 cells (bar = 10 μm). **b** Model of formation or primary nSBs on either chromosome 9, chromosome Y, or both chromosomes 9 and Y. In stressed cell of male origin, pericentric regions of either chromosome 9, chromosome Y, or both are actively transcribed. The transcription of *SATIII* repetitive sequences at nSBs results in the titration of transcription and splicing factors from the rest of the nucleus

In female cells, heat shock leads to HSF1 binding to chromosome 9 *SATIII*-rich genomic region, which in turn activates transcription of the region. *SATIII* RNA accumulation allows the massive recruitment of splicing factors to the nascent RNA SATIII (Biamonti and Vourc’h 2010). We thus checked whether HSF1-activated transcription of the Yq12 genomic region also led to the recruitment of RNA splicing factors such as the SR proteins. Indeed, as shown in Fig. 4a, in HT1080 cells, the SR protein SRSF1 (SF2/ASF) is recruited in a heat shock-dependent manner to chromosome Y (Metz et al. 2004). This later result indicates that SATIII transcripts from chromosome Y are able to direct the formation of functional nSBs. Hence, in male cells, functional nSBs form at either chromosome 9, chromosome Y, or both.

## Discussion

Until now, chromosome 9 has been considered in the literature to be the primary target of HSF1, while several secondary nSBs have been reported on other chromosomes enriched in *SATII* and *SATIII* sequences (Eymery et al. 2010). We now bring evidence that chromosome Y, and more specifically chromosome Y-specific *SATIII* sequences, also constitute a primary target for HSF1 in male human cells and that these sequences are actively transcribed upon heat shock. The presence of canonical HSEs on *SATIII* sequences of chromosome Y, together with our finding that HSF1 is recruited to these sequences upon heat shock, indicates that, as for chromosome 9, chromosome Y-specific *SATIII* DNA repeats are binding sites for HSF1 in response to stress. The reason why, in HT1080 cells, primary nSBs exclusively form on chromosome Y, and not on chromosome 9, as in all other normal and tumor cells investigated so far, is intriguing. Clearly, the 9q12 region in HT1080 cells still displays a capacity to bind HSF1 in in vitro experiments in which HT1080 mitotic chromosome spreads were incubated with purified HSF1 (Fig. 2a). Our in vivo data suggest that the absence of HSF1 binding to the 9q12 region in HT1080 cells relies on a lower affinity of HSF1 for the 9q12 region compared with the Yq12 region, since binding to 9q12 does occur when HSF1 is overexpressed. Accordingly, in normal male fibroblasts, this lower affinity of HSF1 to chromosome 9, compared with chromosome Y, could explain why, despite the presence of two chromosome 9 and of a unique chromosome Y, the same percentage of cells displaying nSBs at chromosome Y and 9 is observed (Fig. 1c). A possible explanation for this differential affinity could be the presence of more HSEs at Yq12 compared with 9q12. Alternatively, it is also possible that in the case of H1080 or H460 cells, a cofactor is either assisting HSF1 toward chromosome Y targeting or, alternatively, reducing HSF1 binding to chromosome 9. The possibility of polymorphisms affecting the HSEs present on 9q12 or Yq12 themselves is unlikely given the large number of repetitive motifs, thus limiting the impact of a few mutational events at the Yq12 locus. Intriguingly, on the basis of a recent article showing that cis accumulation of *SATIII* transcripts causes the mis-segregation of the chromosome from which they originate (Giordano et al. 2020), the expression of *SATIII* RNAs and formation of primary nSBs on chromosome Y may represent a significant source of instability for this chromosome too. However, while we were able to confirm the large percentage of chromosome lagging affecting chromosome 9 on HeLa cells used as a control, we were not able to detect chromosome instability in H1080 cell line submitted to heat shock followed or not to different time of recovery at 37 °C (data not shown). The reasons underlying these differences are still unclear and need further investigations.

In conclusion, our study reveals that *SATIII* sequences of both chromosome 9 and Y can be primary targets of HSF1 (Fig. 4b). Regardless of the exact mechanisms underlying the selectivity of nSBs formation on either the 9q12 or Yq12 regions, we have found that the expression level of HSF1 is an important variable in this selectivity of binding. Although we cannot exclude that an enrichment in acetylated histone marks, at the pericentric regions of either chromosome 9 or Y, may have a direct impact on chromatin cohesion in stressed cells, this study strongly supports the idea that, the transcription of *SATIII* DNA, resulting in the accumulation of *SATIII* RNA and of RNA binding factors at the site of transcription (Vourc'h and Biamonti 2011; Col et al. 2017) are the key determinants in the heat shock response that may act as, or be part of, a checkpoint to preserve genome integrity and prevent cell progression into mitosis (Jolly and Lakhotia 2006; Biamonti and Vourc’h 2010). Our study strongly supports the fact that the exact nature and location of the *SATIII*-enriched genomic region targeted by HSF1 and forming a primary nSB are rather accessory.

## Material and methods

### Cell culture and heat shock treatment

HeLa, HT1080, and male amniocyte cells (Grenoble CHU) were grown in DMEM 10% SVF and McCoy's 5A (Gibco™) 10% SVF, respectively. Normal skin fibroblasts (Coriell Institute, USA) were grown in EMEM 15% SVF. Rodent-human somatic cell hybrids (Antonacci et al. 1995) were grown in RPMI 10% fetal bovine serum, 50 μg/mL gentamicin, and 2 mM L-glutamine.

For FISH and immunofluorescence experiments, cells were grown on glass slides. Metaphase spreads were prepared from HT1080 cells according to standard cytogenetic techniques. Heat shock was performed by immersing the slides for 1 h (HT1080 cells and normal fibroblasts and amniocytes) or 30 min in a water-bath set at the desired temperature (43 °C for tumor cell lines, 45 °C for primary cells).

### Immunofluorescence, DNA FISH, and RNA FISH

Detection of HSF1 by immunofluorescence was performed on 4% formaldehyde-fixed cells as previously described (Jolly et al. 2004). Rabbit anti-HSF1 antibodies 1:250 (Cell signaling) were detected with Goat Anti-Rabbit Dylight 549 nm 1:500 (Vector Laboratories, Inc.).

Probes specific for the *SATIII* sequences of chromosome 9 (pHuR98) (Grady et al. 1992), for chromosome Y (Cooke et al. 1982) or for alphoid sequences of chromosome 9 (pMR9A) (Rocchi et al. 1991), for *SATIII* sequences (cy3-labeled oligonucleotides 5’-ATTCCATTCCATTCCATTCCATTCCATTCCA-TTCCATT-CCATTCCATTCC-3’, were used. Samples were mounted with a Vectashield mounting medium (Vector Laboratories, Inc.) containing 250 ng/μL DAPI.

Combined DNA FISH and immunofluorescence was performed as follows: immunofluorescence was first performed on formaldehyde-fixed cells. After detection with the secondary antibody cells were fixed again in 4% formaldehyde for 10 min, and processed for DNA FISH on ethanol dehydrated cells. Briefly, 200 ng of biotin-labeled probe was precipitated together with 10 μg of salmon sperm DNA, and resuspended in 50% formamide/2× SSC/20% dextran sulfate, and added on the slide. Samples were denatured 5 min on a heating plate at 86 °C, and hybridized overnight at 37 °C. Probes were detected with streptavidin-Alexa 488 or anti-dig alexa 486 (Molecular probes).

RNA was performed as follows: cells were fixed in 4% formaldehyde for 10 min, incubated for 20 min in 20% glycerol/PBS, permeabilized by four successive freezing–thawing cycles in liquid nitrogen, and subsequently dehydrated and hybridized.

Images were acquired with a Zeiss apotome microscope using the 63×, 1.25 NA oil immersion objective. Alternatively, when immunofluorescence signals were not altered by the DNA FISH procedure, DNA FISH and immunofluorescence signals were imaged at the same time.

In vitro reconstitution of HSF1 foci on mitotic from HT1080 cells was performed as previously described (Eymery et al. 2010). Metaphase chromosome spreads were incubated at 37 °C for 3 h with 100 ng of recombinant human HSF1 protein (Stressgen, Assay Designs Inc.) resuspended in Hepes 50 mM pH 7.4, EDTA 0.1 mM, NaCl 200 mM. Slides were subsequently processed for immunofluorescence as described above.

### Chromatin immunoprecipitation

ChIP were performed with a rabbit anti-HSF1 antibody at a concentration of 300 ng/μL. A rabbit antibody against IgG was used as a control. Chromatin fractions (500 bp fragments) were then incubated for 4 h at 4 °C with magnetic beads coupled to protein G (Stressgen). Beads were pelleted and then successively washed for 5 min at 4 °C on a wheel with a nuclear lysis buffer (50 mM Hepes pH 7.9/140 mM NaCl/1 mM EDTA/1% Triton X-100/0.1% Na deoxycholate/0.1% SDS) with a nuclear lysis buffer containing 0.5 M NaCl (2×), with a washing buffer (20 mM Tris-HCl pH(8)/250 nM LiCl/1 mM EDTA/0.5% NP-40) (2×), and finally with an elution buffer Tris-HCl pH(8)/1 mM EDTA (1×). Quantitative PCR were then performed on the precipitated DNA fractions with the Sybergreen kit (Roche) with the following set of probes: the *HSP*70 promoter gene (Rev: 5′-CCCTGGGCTTTTATAAGTCG-3′; for: 5′-GAAGACTCTGGAGAGTTCTG-3′), the *GAPDH* promoter gene (Rev: 5′-ATGGTTGCCACTGGGGATCT-3′; for: 5′-TGCCAAAGCCTAGGGGAAGA-3′) and the Yq12 region: (Rev: 5′-GAGTCAATTCCTTTCGACACCC-3′; for: 5′-TGGACAGGCCTGGAATAAAGTGAA-3′). ChIP experiments were performed on three independent experiments.

### Western blot

Ten micrograms of whole protein extracts prepared in 8 M urea were fractionated by SDS-8% polyacrylamide gel electrophoresis (SDS PAGE). Western blots were performed with anti-HSF1 (1:1000; Enzo Life Sciences, ADI-SPA-901) and anti-tubulin (1:1000; Sigma, T5168). Following washes, membranes were incubated with secondary horseradish peroxidase-conjugated anti-rabbit or anti-mouse antibodies (1:5000; GE Healthcare). Detection was performed with a Biorad Chemidoc and quantification was performed with ImageJ.

## Supplementary information

Fig. S1 Purified HSF1 binds to chromosome 9q12 regions in in vitro binding assays on mitotic spreads of HT1080 cells. HSF1 (in red) is detected by immuno-fluorescence. HSF1 is detected at the 9q12 locus (green signal) detected by FISH (arrows). (TIFF 1.98 mb)

High resolution image (PNG 460 kb)

Fig. S2 HSF1 target chromosomes Y on the H460 tumor cell line. HSF1 is detected by immunodetection (green signal) together with chromosome Y (Cooke et al. 1982) (red signal) by DNA FISH. H460 cells are diploid for chromosome Y. 79% of HSF1 foci are present on chromosome Y (bar = 5 μm). (PPTX 1.95 mb)

Fig. S3 HSF1 is expressed at a similar level in both HeLa and HT1080 cells. 10 μg of whole cellular protein extracts from HeLa and HT1080 cells were submitted to a SDS-PAGE and analyzed by western blot. A similar amount of HSF1 is detected in both cell lines (P-HSF1 = Phospho-HSF1 = DNA-binding competent HSF1 fraction). (PPTX 717 kb)

Fig. S4 HSF1 targets *SATIII* of chromosome Y in HT1080 cells. **a** 426 bp sequence of *SATIII* sequence from the DYZ1 clone specific for the Yq12 region (Nakahori et al. 1986) with the positions of the two primers (underlined sequence) and the position of the putative HFS1 binding site (framed sequence). **b** Fusion curves obtained in the qPCR reaction with oligos specific for *HSP*70 and Y-specific *SATIII* sequences is shown. Profiles of Yq12 and *HSP*70 qPCR amplification melt curves for *HSP*70 and Yq12. **c** Western blots anti-HSF1 from anti-HSF1 ChIP experiments. Samples were run on a SDS/PAGE 8% acrylamide gel (HS1-P = phospho HSF1). (PPTX 1.96 mb)
