# Supplementary figures and images for: Chromosome Y pericentric heterochromatin is a primary target of HSF1 in male cells
Source: Chromosoma. 2021 Feb 6;130(1):53–60. doi: 10.1007/s00412-021-00751-2 (PMC7889540; doi:10.1007/s00412-021-00751-2)

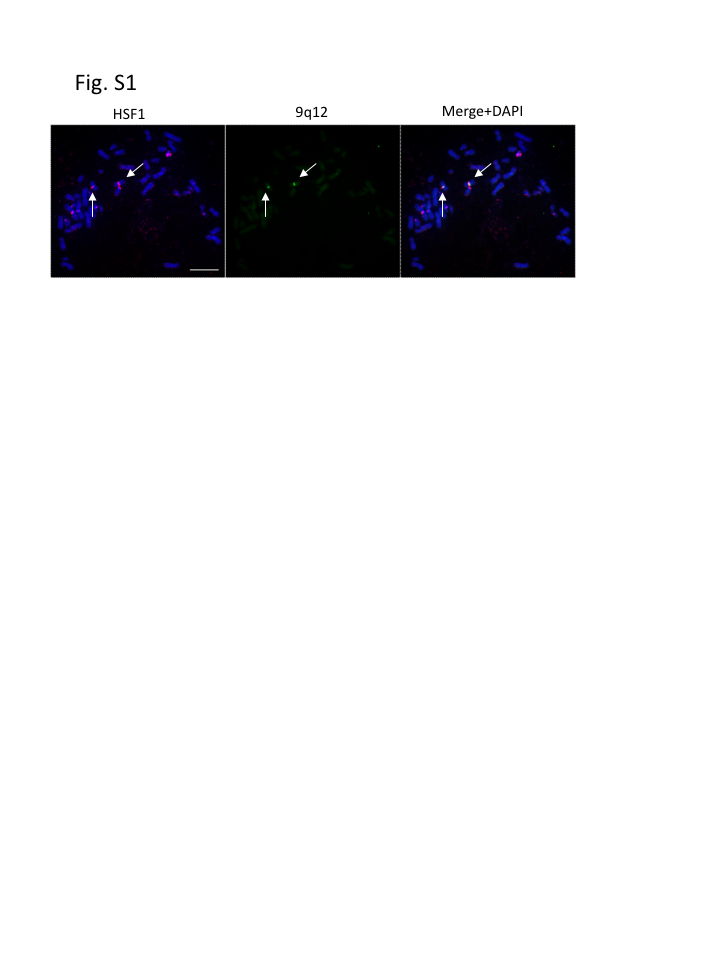

Supplement: Supplementary file 1 — Purified HSF1 binds to chromosome 9q12 regions in in vitro binding assays on mitotic spreads of HT1080 cells. HSF1 (in red) is detected by immuno-fluorescence. HSF1 is detected at the 9q12 locus (green signal) detected by FISH (arrows). (TIFF 1.98 mb) [file 412_2021_751_MOESM1_ESM.tiff]

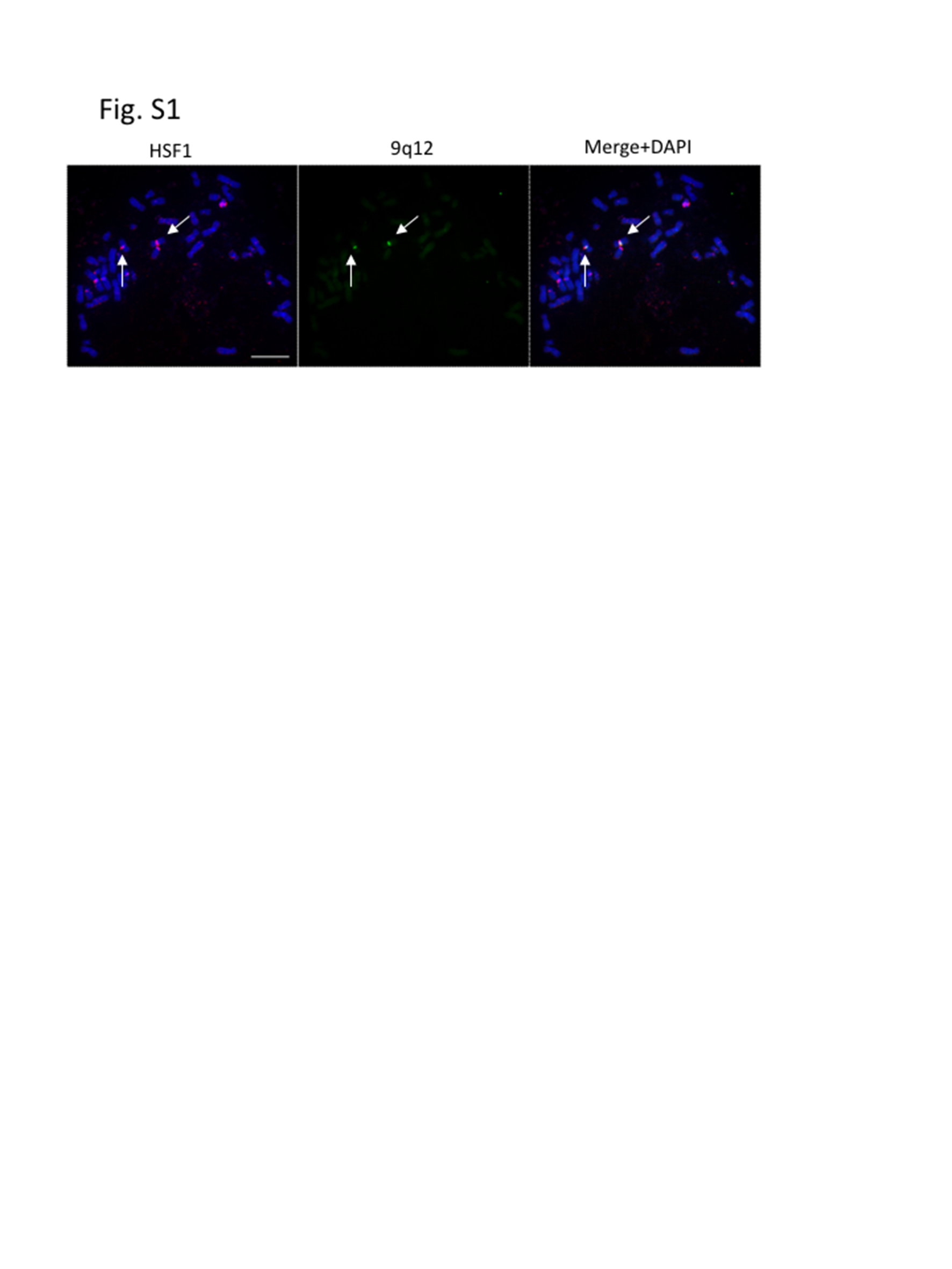

Supplement: Supplementary file 2 — High resolution image (PNG 460 kb) [file 412_2021_751_Fig5_ESM.png]

## Slide 1
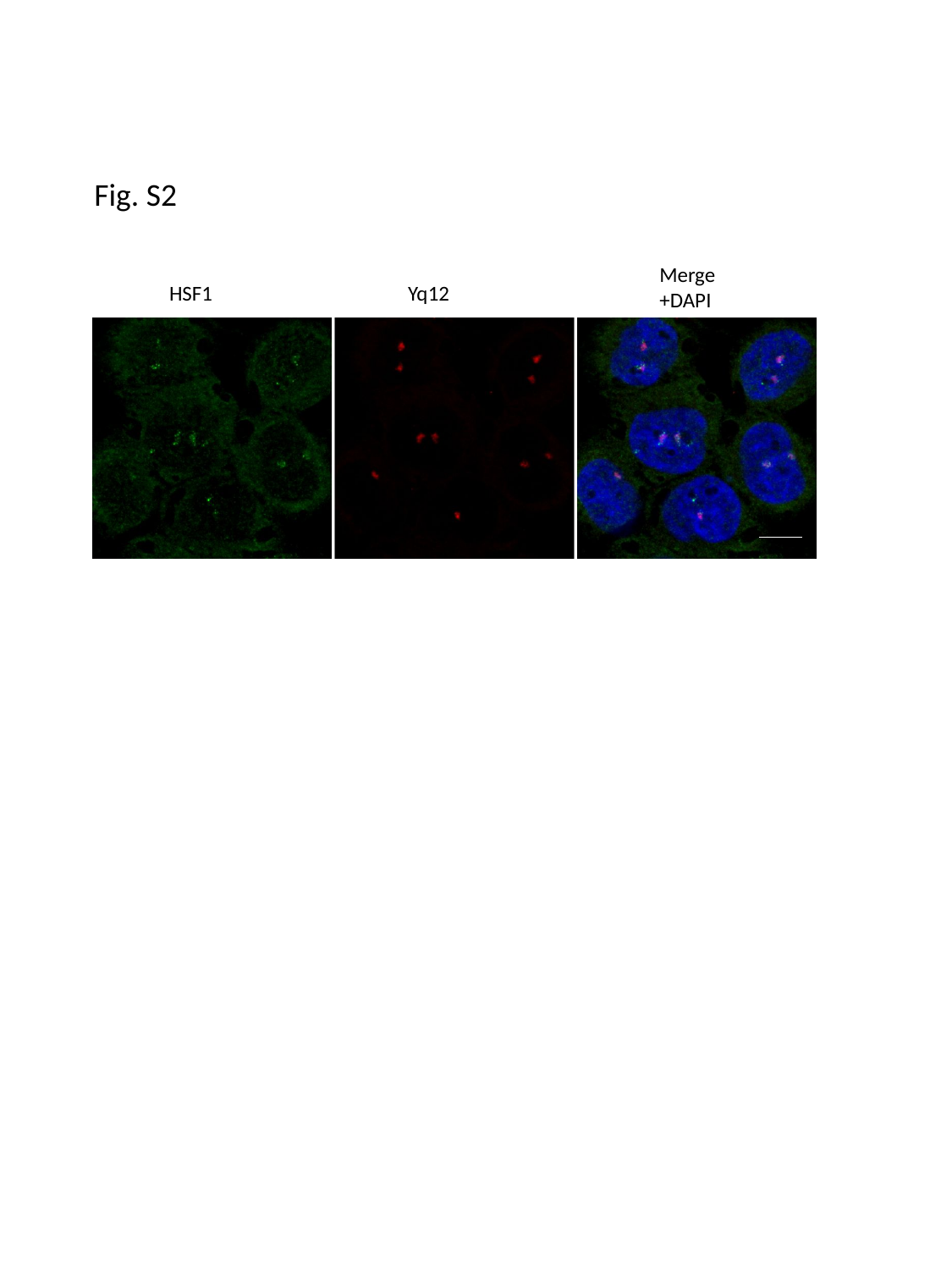

Fig. S2
Merge
+DAPI
HSF1
Yq12

Supplement: Supplementary file 3 — HSF1 target chromosomes Y on the H460 tumor cell line. HSF1 is detected by immunodetection (green signal) together with chromosome Y (Cooke et al. 1982) (red signal) by DNA FISH. H460 cells are diploid for chromosome Y. 79% of HSF1 foci are present on chromosome Y (bar = 5 μm). (PPTX 1.95 mb) [file 412_2021_751_MOESM2_ESM.pptx]

## Slide 1
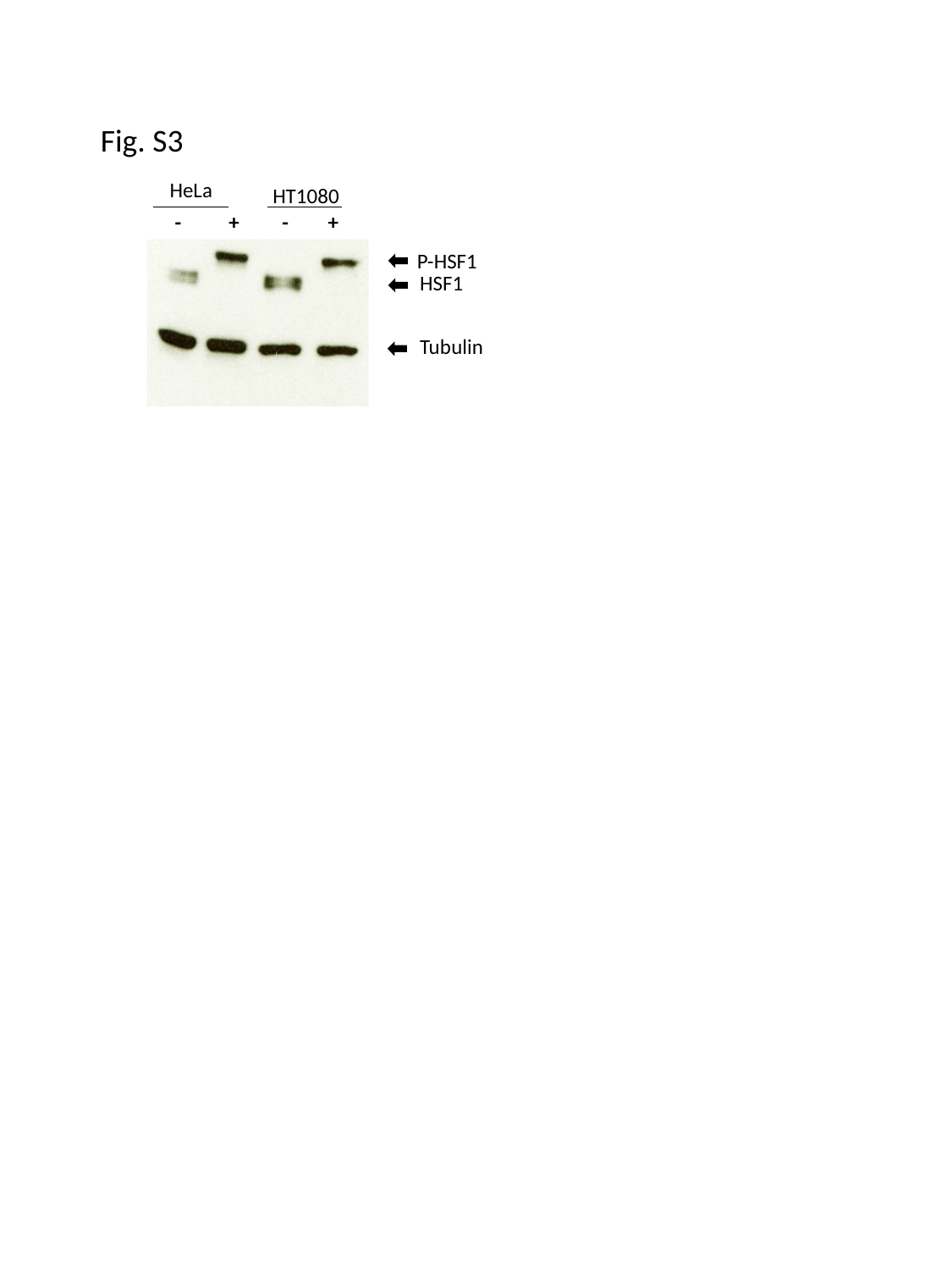

Fig. S3
HeLa
HT1080
-
+
-
+
P-HSF1
HSF1
Tubulin

Supplement: Supplementary file 4 — HSF1 is expressed at a similar level in both HeLa and HT1080 cells. 10 μg of whole cellular protein extracts from HeLa and HT1080 cells were submitted to a SDS-PAGE and analyzed by western blot. A similar amount of HSF1 is detected in both cell lines (P-HSF1 = Phospho-HSF1 = DNA-binding competent HSF1 fraction). (PPTX 717 kb) [file 412_2021_751_MOESM3_ESM.pptx]
