# Supplementary material for: Chromosome Y pericentric heterochromatin is a primary target of HSF1 in male cells
Source: Chromosoma. 2021 Feb 6;130(1):53–60. doi: 10.1007/s00412-021-00751-2 (PMC7889540; doi:10.1007/s00412-021-00751-2)
Supplement: Supplementary file 5 — HSF1 targets SATIII of chromosome Y in HT1080 cells. a 426 bp sequence of SATIII sequence from the DYZ1 clone specific for the Yq12 region (Nakahori et al. 1986) with the positions of the two primers (underlined sequence) and the position of the putative HFS1 binding site (framed sequence). b Fusion curves obtained in the qPCR reaction with oligos specific for HSP70 and Y-specific SATIII sequences is shown. Profiles of Yq12 and HSP70 qPCR amplification melt curves for HSP70 and Yq12. c Western blots anti-HSF1 from anti-HSF1 ChIP experiments. Samples were run on a SDS/PAGE 8% acrylamide gel (HS1-P = phospho HSF1). (PPTX 1.96 mb) [file 412_2021_751_MOESM4_ESM.pptx]

## Slide 1
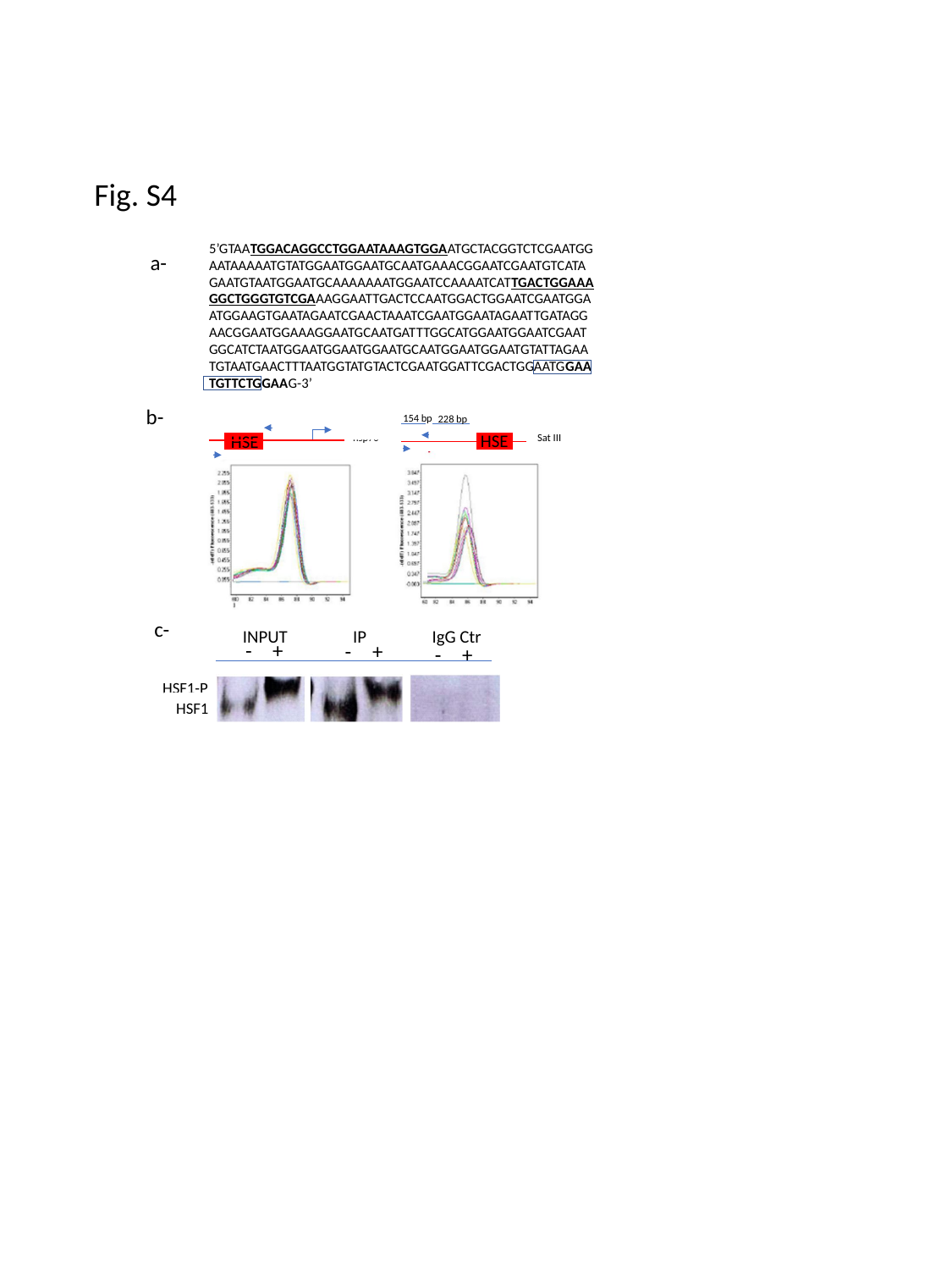

Fig. S4
5’GTAATGGACAGGCCTGGAATAAAGTGGAATGCTACGGTCTCGAATGGAATAAAAATGTATGGAATGGAATGCAATGAAACGGAATCGAATGTCATAGAATGTAATGGAATGCAAAAAAATGGAATCCAAAATCATTGACTGGAAAGGCTGGGTGTCGAAAGGAATTGACTCCAATGGACTGGAATCGAATGGAATGGAAGTGAATAGAATCGAACTAAATCGAATGGAATAGAATTGATAGGAACGGAATGGAAAGGAATGCAATGATTTGGCATGGAATGGAATCGAATGGCATCTAATGGAATGGAATGGAATGCAATGGAATGGAATGTATTAGAATGTAATGAACTTTAATGGTATGTACTCGAATGGATTCGACTGGAATGGAATGTTCTGGAAG-3’
a-
b-
154 bp
228 bp
HSE
Sat III
HSE
hsp70
c-
INPUT
IP
IgG Ctr
-
+
-
+
-
+
HSF1-P
HSF1
